# Motion attenuation surgery in the degenerative lumbar spine: Is cement discoplasty a safe and effective option?

**DOI:** 10.1016/j.bas.2025.104220

**Published:** 2025-03-04

**Authors:** Derek T. Cawley

**Affiliations:** a University of Galway, Ireland; b Mater Private Hospital, Dublin, Ireland

**Keywords:** Cement discoplasty, Polymethylmethacrylate, Intradiscal vacuum phenomenon, Adjacent segment degeneration, Facet arthrosis, Degenerative scoliosis

## Abstract

Cement discoplasty (CD) was initially described in 2015. This novel treatment involves injecting polymethylmethacrylate (PMMA) into the degenerate disc cavity. This is particularly applicable to elderly patients with disc degeneration and collapse, where restoration of disc height improves lordosis and sagittal balance, treating symptoms of degenerative scoliosis, foraminal stenosis, adjacent segment degeneration, or flatback syndrome, who would otherwise have significant risks for major spine surgery. In all cases, symptoms are associated with intradiscal vacuum phenomenon (IDVP), a radiological finding associated with advanced disc degeneration. The technique is neither a motion preserving nor fusion procedure. While cement acts best in compression, the concept of stabilising but not fusing the spine in such cases lacks certainty and clarity as to its clinical effectiveness. This narrative review discusses the concepts of this technique, 12 clinical series and four metanalyses, mostly advocating for its use, particularly where it delivers a solution with an acceptable safety profile, short length of stay and short recovery time.

## Highlights

• Over 12 clinical series have been published along with four metanalyses, mostly advocating for its use, particularly where it delivers a solution with an acceptable safety profile, short length of stay and short recovery time.

## 1 Introduction

Cement applications to spinal pathology have endured since Deramond of Amiens, France described intravertebral injection of PMMA (polymethylmethacrylate) for a painful haemangioma in 1984 (Galibert et al., 1987).

Cement discoplasty (CD) involves injecting polymethylmethacrylate (PMMA) into the degenerate disc, where when positioned prone, it assumes the shape of the cavity. The minimalist aspect of this treatment is particularly applicable to elderly patients with disc degeneration and collapse, where restoration of disc height improves lordosis and sagittal balance, treating symptoms of degenerative scoliosis, foraminal stenosis, adjacent segment degeneration, or flatback syndrome, who would otherwise have significant risks for major spine surgery. The ensuing disc height restoration confers a stretch on the annulus fibrosis allowing ligamentotactic-type stability. In all cases, symptoms are associated with intradiscal vacuum phenomenon (IDVP), a radiological finding associated with known causes of disc degeneration in those over 60 years of age (Murata et al., 2018; Kanna et al., 2022; Ekşi et al., 2022; Camino-Willhuber et al., 2023; Cawley et al., 2024). The technique is used without additional fixation, or may complement pedicle screw constructs particularly in cases of rotational or translational instability, or where the edge-loading of an intradiscal cage is otherwise unreliable. While cement acts best in compression, the concept of stabilising but not fusing the spine in such cases lacks certainty and clarity as to its clinical effectiveness.

Motion attenuation as a concept has not been prevalent in spine surgery, whereas motion preservation is prominent, largely including disc arthroplasty techniques. Motion attenuation may be observed in cases of post-instrumentation asymptomatic pseudarthrosis, where relative or sufficient spinal stability has been achieved. While solid fusion correlates with optimal surgical outcomes, pseudarthrosis does not to the same extent. Sufficient stability, while mostly sub-optimal may in some cases yields significant clinical improvements (Djurasovic et al., 2011; Herkowitz and Kurz, 1991; Tokuhashi et al., 2008), correction of the observed excessive degenerative motion (narrowing of the neutral zone) may decrease the associated symptoms of low back pain (Yue et al., 2007). Without the morbidity of large surgery, it is in this context that CD may provide benefit. AO (Arbeitsgemeinschaft für Osteosynthesefragen) concepts regarding fixation of the spine include stability, alignment, biology and function. Interestingly, from AO came the principle of relative, as opposed to absolute stability which may find relevance with CD.

## 2 Clinical evidence

Cement discoplasty was initially described as the result of a vertebroplasty procedure, where the adjacent disc with vacuum phenomenon filled with PMMA, and signs of instability had disappeared postoperatively (Varga et al., 2015). Varga et al. included 81 patients, treated for foraminal stenosis. Patients reported a reduction in their lower back and leg pain (69% and 66%) postoperatively and 61% of patients had a minimum 10-point reduction in their ODI scores at six months. At 6-month follow-up, 61% of patients had a minimum 10-point reduction in their ODI scores (p < .01). The clinical results of percutaneous CD were immediate, without long hospitalization. Additional work by the same group outlined significant improvements in lumbopelvic parameters (sacral slope, segmental scoliosis, disc height) with clinical outcomes (ODI, low back pain and leg pain) (Kiss et al., 2019). Similar to observations from lumbar decompression, overall lumbar lordosis (LL) did not significantly change (3.4°, p > .05), but the pre-to post-op to six-month segmental lordosis (4.4° ± 3.8° vs. 6.6° ± 4.8° vs. 6.9° ± 4.7°) showed significant, repeated changes after the procedure (p < .05). Similar improvements were seen in coronal alignment. Interestingly, improvements in leg pain (−1.8 ± 2.5 vs. − 3.2 ± 2.3, p < .05) were significantly greater in multilevel procedures, presumably because of the cumulative correctional effect.

Yamada et al. reported on degenerative scoliosis patients, at around the same time, treating 109 patients with CD and 53 with nonoperative treatment. Mean improvements in VAS scores were 52.2 and 4.0 and improvements in ODI were 20.7 and 1.0 for CD and nonoperative groups, respectively. Low back pain recurred in 13 (13%), requiring additional CD. Yamada et al. paid particularly attention to bone marrow oedema on the concave aspect of the degenerative curve that correlated with LBP. This reappeared at the initial level in six patients and newly appeared at the adjacent level of CD in seven patients, where they repeated CD and which again improved VAS ODI at 2 years (Yamada et al., 2016, 2021; Nakamae et al., 2022). All clinical reports are largely confined to a follow up of two years or less, which may be acceptable in an elderly or frail population. However, recurrence of endplate oedema and likely micro-fracturing as outlined by Yamada et al. outlined a potential long term evolutionary outcome.

Camino-Willhuber et al. followed up outcomes of 54 patients with back pain, with advanced disc disease and with/without degenerative scoliosis for over one year (Camino et al., 2020). They observed significant pain reduction (VAS change: 7.8 to 4.4), ODI improvement (62–36.2) and partial correction of radiological parameters (5° mean increase in lumbar lordosis and 5° decrease in Cobb angle) at one year. Mean surgical time was 38 min, and the mean length of hospital stay was 1.2 days. Similarly Dittmar-Johnson et al. described CD as treatment for six cases with severe disc degeneration with IDVP, noting an improvement in five patients (VAS mean 5.6) (Michael et al., 2022). Tian et al. took an anti-inflammatory approach, using CD for filling a discectomy void to treat endplate osteochondritis/Modic 1 changes (Tian et al., 2017). Clinical series outlined in Table 1.

**Table 1:** Reported clinical series published in English, German and Spanish. ∗∗ approximate.

Study, Year	Participants with follow up/total	Diagnosis	F/Up (months)	Outcomes	Surgery Time (mins)/Length of Stay (days)	Complications
Varga et al., 2015	47/81	Disc Degeneration	6	VAS 6.8 to 4.3, 61% > 10-pt decrease in ODI	25 (1 level)	No post-operative complications reported
Yamada et al., 2016	109/162	Degenerative scoliosis	24	Improvements VAS -52.2 and ODI -20.7	38/-	Cement leakage 3.13 had recurrence of BME with pain, of which 4/13 had repeat procedure and subsequent relief
Yamada et al., 2021	80/101	Degenerative scoliosis	>24	VAS 82 to 43, ODI 51 to 35, Cobb 18 to 16	–	As above
Nakamae et al., 2022	40/40	Degenerative scoliosis	Mean 24, Min 6	Cobb 16 to 10	28/-	No major intraoperative and postoperative complications, including cement leakage.
Kiss et al., 2019	63/28	Disc Degeneration	6	VAS Back 5.9 to 3.5, VASS Leg 6.9 to 2.4, ODI 55.4 to 37.9. SL 4.4 to 6.9, Cobb 7.4 to 5.7	22.5 (1 level)/-	No post-operative complications reported
Camino-Willhuber 2020,	82/54	37 Degenerative scoliosis, 17 disc degeneration	>12	VAS 7.8 to 4.4, ODI 62 to 36.5° increase in LL and 5° decrease in Cobb	38/1.2	4 decompression, 1 deep infection, 1 adjacent fracture
Camino-Willhuber, 2021	180/156	67 Degenerative scoliosis; 29 disc degeneration; 15 adjacent segment disease; 14 stenosis; 12 spondylolisthesis; 5 non-union; 12 combined pathologies	>24	VAS 7.8 to 3.6 ODI 68.1 to 17.2	-/1.4	5.7%: 5 decompression surgery; 2 deep infections; 1 deep vein thrombosis; 1 adjacent vertebral fracture; 15 unplanned readmission within 30 days that required intravenous medication; 5 cases required PCD in another level due to pain recurrence; 4 cases required fusion surgery due to persistent pain
Dittmar-Johnson, 2022	6/6	5 disc degeneration; 1 spondylolisthesis	6	VAS 8.4 to 2.8	−/−	1 conversion to fusion
Tian et al., 2017	7/7	Endplate osteochondritis & disc herniation	12	VAS Back/Leg 6.1/7.3 to 1.7/1.9. ODI 77 to 19.	55/7.5	1 leakage without clinical signs
Tian, 2019	16/16	Endplate osteochondritis & disc herniation	12	VAS 6.8/7 to 2.4/2.4 ODI	−/−	2 cement leakages (symptoms disappeared within 24 h s without treatment)
Eltes, 2021	10/10	Disc degeneration	6	Improvements VAS Back/Leg-16/-14, ODI -24	−/−	No post-operative complications reported
Koch et al., 2023	80/344	Disc degeneration	>12	VAS -1.7∗∗, ODI -15, LL increase 5° SS	30/3	30% cement leakage - 6.4% symptomatic, 54 (15%) required additional surgery: decompression 31, instability 12, adjacent segment 8, fracture 3.

Abbreviations: F/up: Follow up, VAS:Visual Analogue Score, ODI: Oswestry Disability Index, LL: Lumbar lordosis, mins: minutes, hrs: hours, Min: minimum.PCD: Percutaneous cement discoplasty.

Four systematic reviews specifically on CD have recently been published. Techens et al. included 12 clinical series where it “consistently reported that PCD significantly improved the clinical status of the patients and maintained it after two years” (Techens et al., 2022a). It recognised that all papers “acknowledged their risks of bias and tried to mitigate them”. Grewal et al. reviewed six papers (336 patients), excluding smaller or overlapping series, with similar conclusions (Grewal et al., 2023). They noted a total complication rate of <15%. They recognised methodological limitations and a high risk of bias, that the validity and generalizability of the findings were uncertain, but concluded “the results provide preliminary insights into PCD's potential efficacy and can guide future research to address current limitations.” The papers in this review with suggested bias, were by Varga et al. (2015) (the original study with risk of confounding and selection bias) and by Alkharsawi et al. (2022) (CD for salvage of pseudarthrosis post-instrumented fusion – largely irrelevant). Salvage cement augmentation of existing instrumentation has also been described separately (Cawley et al., 2023). Similar findings were made by Fusini and Zhang et al. (Fusini et al., 2022; Zhang et al., 2024).

## 3 Safety profile

The safety profile was evaluated specifically for CD (Techens et al., 2022a; Grewal et al., 2023). It is acknowledged that the morbidity of a percutaneous procedure is decreased compared to open surgery, not least the alternative of instrumented fusion, particularly in the setting of degenerative scoliosis or extension of previous fusion, or for elderly patients. Cement leakage in the intervertebral foramen and vertebral body fracture were the most common (<5% and <1%). Koch et al. subsequently analysed complications of CD in a cohort of 344 patients, reporting a cement leakage in 30.4% of which 20% were symptomatic (leg pain 5.2%, motor deficit 1.2% and sensory deficit 2.6%, other 2%), without affecting length of stay or ultimate outcome. They noted age, subcutaneous fat tissue thickness, low viscosity cement, lower level of surgeon's experience and the number of operated levels as risk factors of cement leakage (Koch et al., 2023).

## 4 Biomechanical studies

Reporting on biomechanical studies has significantly increased since 2022 and have included cadaveric analysis, computational models and studies on cement design. Increases in disc height and foraminal opening have been achieved in animal (Techens et al., 2020; Ghandour et al., 2022) and human cadaveric (Techens et al., 2022b) models to those observed clinically. The neutral zones in flexion–extension and lateral bending were significantly reduced in a porcine (non-degenerate) model, treated with CD, with persistence of some translation of the cement and fracture of the cement at the insertion pedestal along the annulus puncture channel (Huang et al., 2022). Simulations of human cadaveric analysis with finite element models have also shown increased foraminal height and reductions in segmental kinematics, particularly in flexion. While also simulating disc degeneration with nerve root stress, increases were observed followed by decreases after CD (Jia et al., 2022).

In a goat model, incorporation of osteogenic mineralised collagen to PMMA improved hydrophilicity, with reductions of fibrous tissue encapsulating the injected amount within the disc space. After three months, there was also an increased circumferential contact with bone (Yang et al., 2020). The current standard PMMA cements, which have so far been used in discoplasty, have a much higher elastic modulus (∼3000 MPa) than that of the surrounding vertebral bone (10–900 MPa) (Morgan et al., 2003; Nazarian et al., 2008; Crawford et al., 2003; Helgason et al., 2008). Lower modulus PMMA has been simulated to show reduced end-plate stress when compared to standard PMMA (Lewin et al., 2022). Linoleic acid has been used as a plasticiser to lower the modulus to approximate bone and may be applicable (Robo et al., 2021).

## 5 Patient selection

**Fig. 1:**
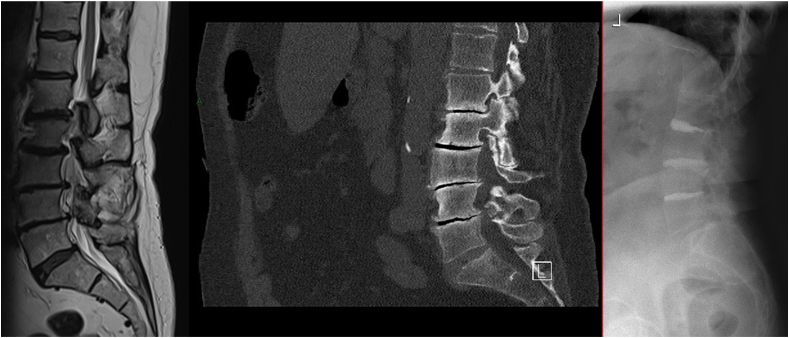
72 year old woman with severe arthritic back pain, bilateral (L5) neurogenic leg pain, hamstring fatigue, poor mobility, high BMI and diabetes. After initial rehabilitation and attempted weight loss, she was treated with L34 & L45 decompression and cement discoplasty (CD) at L23, L34 & L45. No peri-operative or early complications. A: Pre-operative mid-sagittal MRI image displaying multilevel disc degeneration & stenosis. B: Mid-sagittal CT image indicating large abdominal girth and multilevel intradiscal vacuum phenomenon (IDVP) and sclerotic endplates. C: Follow-up post-operative radiograph with preservation of lumbar lordosis. VAS improved from 8/10 (constant) to 4/10 (episodic) back pain and 0/10 leg pain, and Oswestry Disability Score (ODI) improved from 76% pre-operatively to 36% after two years.

**Fig. 2:**
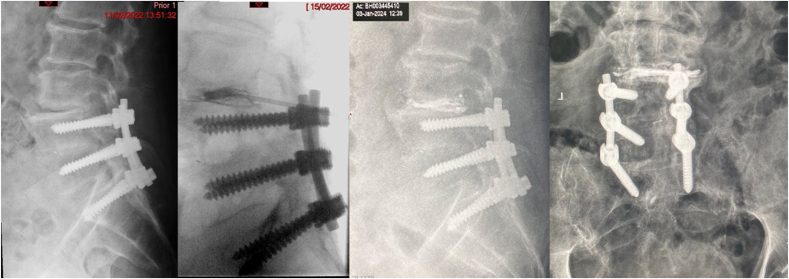
84 year old man with back pain 7/10 and leg pain 5/10, with kidney transplant, early dementia and low BMI, walking-frame reliant, having received L4L5S1 fusion over 15 years previously. Had percutaneous CD. A: Pre-operative radiograph. B: Intra-operative fluoroscopy with trocar in situ, Kambin's triangle approach, at L34 mid-cementation, small cement leak (not in spinal canal or foramena). C & D: Two-year post-operative radiographs. No peri-operative or early complications. VAS had improved to 2/10 in his back, no leg pain, walking stick reliant. ODI had improved from 72% to 28%.

**Fig. 3:**
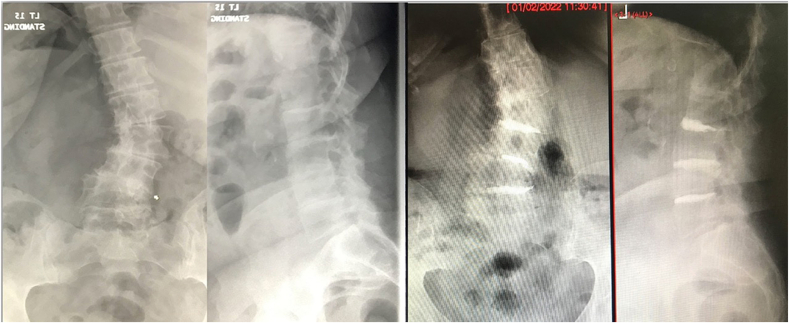
78 year old lady with degenerative scoliosis, back pain 5/10 and leg pain (R L34 dermatome) 9/10. Had L34 extra-foraminal decompression and open CD, percutaneous CD at L23 and L45. No peri-operative or early complications. A & B: Pre-operative radiographs. C & D: Two-year post-operative radiographs with restoration of disc height at affected levels, stabilisation of the scoliosis. VAS was 3/10 and ODI was 22%.

**Fig. 4:**
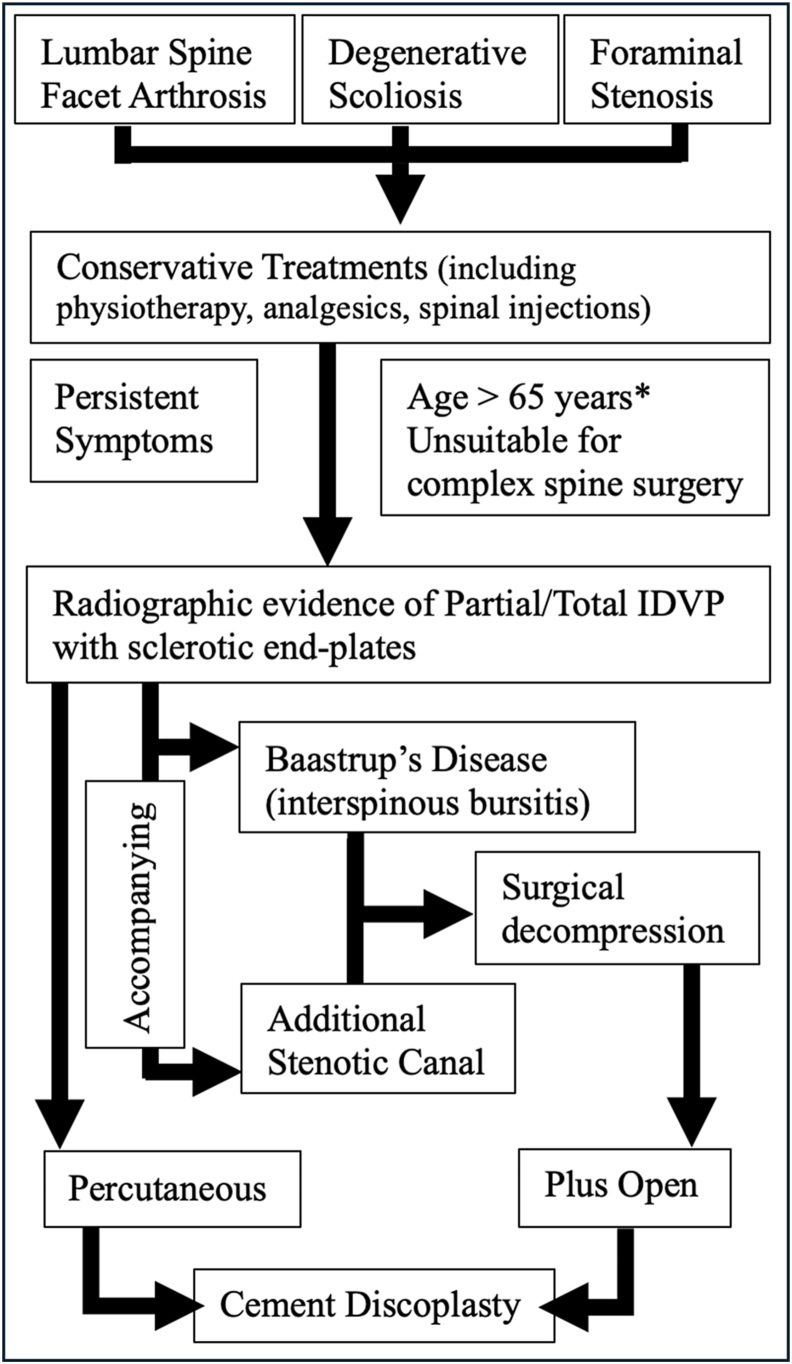
Treatment algorithm for Cement Discoplasty. ∗ Reflective of current evidence.

Typical indications for CD are elderly patients with mechanical low back pain with or without leg pain, vacuum occupying >50% of the disc space, sclerotic endplates, vertical instability characterized by pain aggravating in standing and significant comorbidities that relatively contraindicate more invasive surgeries (Cawley et al., 2023). End plate sclerosis is a strong correlator with evidence of VDP and has been classified by Camino-Willhuber et al. to inform decision making on CD (Camino et al., 2021). They developed a simplified tomographic classification of the air distribution with high reliability. Their outcome series demonstrated that the combination of VDP and endplate sclerosis led to the best outcomes for CD. Standard decompression may be required for lateral recess stenosis and/or Baastrup disease (interspinous bursitis), facilitating open CD. Differentiation of leg and back pain is key (Cawley et al., 2018), as the nociceptive nature of back pain significantly improves but is not as severe nor does it improve with CD to the same extent as neurogenic leg pain (Varga et al., 2015; Kiss et al., 2019). For sagittal or for coronal imbalance indications, greater correction is achieved with at least **t**wo levels of CD (Kiss et al., 2019; Cawley et al., 2023). (Fig. 1, Fig. 2, Fig. 3: Clinical Examples, Fig. 4: Treatment Algorithm).

Contra-indications include subjects who cannot tolerate general anaesthesia, without vacuum on radiograph or CT, and with dynamic translation or translational instability (eg. dynamic spondylolisthesis). Long term follow-up has not yet been reported and due to potential for cement fracture, adjacent vertebral fracture or cement migration, recommendation of 10.13039/100011639CD for patients less than 45 years cannot yet be supported. While treating more caudal levels has greater correctional implications, there is no evidence for CD as a treatment for distal (caudal) adjacent segment degeneration, and likely to fail as the rotational torque on the affected segment is likely too high for CD to achieve sufficient stability and is unlikely to be effective, requiring instrumentation.

## 6 Conclusion

Spinal surgery in elderly or infirm patients is often limited due to comorbidities and has higher complication rates leaving patients instead with conservative and often ineffectual treatments. Cement discoplasty uses established biomechanical concepts, including such as preservation of motion, indirect decompression and anterior column loading, a familiar bio-inert material and reliable access techniques to achieve relative stability and reconstruction of the degenerative disc. Nonetheless, through its minimalist footprint, it displays a safe profile and achieves significant clinical improvements in patients who are often those most difficult to treat.

There was no funding or conflict of interest on behalf of the author in respect of this work.

## Declaration of competing interest

The author has no financial/personal interest or belief that could affect my objectivity, or any source and nature of that potential conflict.
